# Personal, Social, and Game-Related Correlates of Active and Non-Active Gaming Among Dutch Gaming Adolescents: Survey-Based Multivariable, Multilevel Logistic Regression Analyses

**DOI:** 10.2196/games.3092

**Published:** 2014-04-04

**Authors:** Monique Simons, Emely de Vet, Mai JM Chinapaw, Michiel de Boer, Jacob C Seidell, Johannes Brug

**Affiliations:** ^1^EMGO Institute for Health and Care ResearchDepartment of Health SciencesVU University AmsterdamAmsterdamNetherlands; ^2^Body@Work, Research Center Physical Activity, Work and HealthTNO-VU/VUmc, VU University Medical CenterAmsterdamNetherlands; ^3^TNOExpertise Centre Life StyleLeidenNetherlands; ^4^Wageningen University and Research CentreSection Communication, Philosophy and Technology: Centre for Integrative Development, Chairgroup Strategic CommunicationWageningenNetherlands; ^5^EMGO Institute for Health and Care ResearchDepartment of Public and Occupational HealthVU University Medical CenterAmsterdamNetherlands; ^6^EMGO Institute for Health and Care ResearchDepartment of Epidemiology & BiostatisticsVU University Medical CenterAmsterdamNetherlands

**Keywords:** video games, interactive games, active games, adolescent, sedentary lifestyle, physical activity, determinants

## Abstract

**Background:**

Playing video games contributes substantially to sedentary behavior in youth. A new generation of video games—active games—seems to be a promising alternative to sedentary games to promote physical activity and reduce sedentary behavior. At this time, little is known about correlates of active and non-active gaming among adolescents.

**Objective:**

The objective of this study was to examine potential personal, social, and game-related correlates of both active and non-active gaming in adolescents.

**Methods:**

A survey assessing game behavior and potential personal, social, and game-related correlates was conducted among adolescents (12-16 years, N=353) recruited via schools. Multivariable, multilevel logistic regression analyses, adjusted for demographics (age, sex and educational level of adolescents), were conducted to examine personal, social, and game-related correlates of active gaming ≥1 hour per week (h/wk) and non-active gaming >7 h/wk.

**Results:**

Active gaming ≥1 h/wk was significantly associated with a more positive attitude toward active gaming (OR 5.3, CI 2.4-11.8; *P*<.001), a less positive attitude toward non-active games (OR 0.30, CI 0.1-0.6; *P*=.002), a higher score on habit strength regarding gaming (OR 1.9, CI 1.2-3.2; *P*=.008) and having brothers/sisters (OR 6.7, CI 2.6-17.1; *P*<.001) and friends (OR 3.4, CI 1.4-8.4; *P*=.009) who spend more time on active gaming and a little bit lower score on game engagement (OR 0.95, CI 0.91-0.997; *P*=.04). Non-active gaming >7 h/wk was significantly associated with a more positive attitude toward non-active gaming (OR 2.6, CI 1.1-6.3; *P*=.035), a stronger habit regarding gaming (OR 3.0, CI 1.7-5.3; *P*<.001), having friends who spend more time on non-active gaming (OR 3.3, CI 1.46-7.53; *P*=.004), and a more positive image of a non-active gamer (OR 2, CI 1.07–3.75; *P*=.03).

**Conclusions:**

Various factors were significantly associated with active gaming ≥1 h/wk and non-active gaming >7 h/wk. Active gaming is most strongly (negatively) associated with attitude with respect to non-active games, followed by observed active game behavior of brothers and sisters and attitude with respect to active gaming (positive associations). On the other hand, non-active gaming is most strongly associated with observed non-active game behavior of friends, habit strength regarding gaming and attitude toward non-active gaming (positive associations). Habit strength was a correlate of both active and non-active gaming, indicating that both types of gaming are habitual behaviors. Although these results should be interpreted with caution because of the limitations of the study, they do provide preliminary insights into potential correlates of active and non-active gaming that can be used for further research as well as preliminary direction for the development of effective intervention strategies for replacing non-active gaming by active gaming among adolescents.

## Introduction

The prevalence of overweight and obesity in Dutch youth has increased in recent decades [1]. Overweight and obesity represent major public health problems [2,3]. Promoting physical activity (PA) and reducing sedentary behaviors are important targets for the prevention of overweight in youth [4,5].

Playing video games contributes significantly and substantially to sedentary behavior in youth [6-8]. For example, in the Netherlands, 95% of adolescent boys and 85% of adolescent girls play video games for an average of 10 and 4 hours per week (h/wk), respectively [8]. Correlates of playing video games identified previously include gender, age, ethnic background, and parents’ educational level [6,7,9,10]. One of the most consistent findings in these studies is the difference between boys and girls: boys generally spend more time gaming than do girls [6,7-9]. With respect to age, US children aged 11-14 years appear to play more than 8- to 10-year-old children. Regarding ethnic background, Hispanic and African American youth (8-18 years old) spend more time playing video games than white youth [7]. Furthermore, a study among 10- to 11-year-old Flemish children and their parents [9] and a study among 4- to 18-year-old Dutch children and their parents [10] showed that compared to children of higher-educated parents, children of parents with low or medium education levels spend more time playing video games.

A new generation of video games—active games—seems a promising alternative to sedentary games in promoting PA and reducing sedentary behaviors in youth [11-13]. Active games require movement of the body, more than only fingers and hands (eg, Nintendo Wii, PlayStation Move) [14]. Several studies have shown that active games involve light to moderate intensity physical activity (2-6 metabolic equivalents) [11,15], and pilot studies suggest that active gaming is associated with more PA and less sedentary time [13,16]. A large 6-month study showed that playing active games was associated with lower body mass index in children [17]. Furthermore, a number of studies have shown that a substantial proportion of adolescents play active games [17-19]. In the Netherlands, 43% of adolescents indicated that they play active games [19]. In Canada, this percentage was 25% [18].

To our knowledge, little attention has been paid to the correlates and determinants of active gaming. O’Loughlin explored potential sociodemographic, lifestyle, psychosocial, weight-related, and mental health correlates in Canadian adolescents. They showed that active gamers were more likely to be female, play non-active video games, watch ≥2 hours of television per day, be concerned about weight and be nonsmokers compared to adolescents who did not play active games [18]. A Dutch study comparing regular active gamers (≥1 h/wk) with nonregular active gamers (<1 h/wk) showed that regular active gamers (≥1 h/wk) were slightly but significantly younger (13.5 vs 14.1 years old) but did not differ with respect to gender, education level (of adolescent and parent), ethnicity, or sedentary screen time (TV/DVD and computer time) [20].

In a previous study, we examined and compared demographic correlates of active gaming and non-active gaming simultaneously in Dutch adolescents [19]. Irrespective of age, adolescents attending a lower educational level of secondary school were more likely to play active games ≥1 h/wk than adolescents attending higher educational levels. For non-active gaming, gender and age were correlates, with boys and older adolescents being more likely to play non-active games >7 h/wk than girls or younger adolescents [19]. There are no generally accepted cutoff values for active and non-active gaming. Therefore we based the 1 h/wk cutoff value on calculations in adults (because child-specific ones were not available at that time) that demonstrated that excessive weight gain can be prevented if energy balance is affected by 70 kcal/wk [21]. This corresponds with 1 hour of active gaming [15]. Second, 1 h/wk seems feasible to incorporate into intervention programs. Studies show that gaming adolescents spent, on average, 4-10 h/wk playing non-active games [8,19]. Replacing all non-active game time does not seems realistic, but asking to replace 1 hour out of 4-10 hours seems doable. Furthermore, only 28% of the active gaming adolescents played for ≥1 h/wk, indicating there is still room for improvement [19]. For non-active gaming, we used 1 hour per day (7 h/wk) as the cutoff value because this represents half of the 2 hours of maximum total screen time recommended for adolescents [22] and is the same cutoff value used by Allahverdipour et al in their study on non-active gaming [23].

In summary, previous studies tended to focus on the prevalence and on demographic characteristics of (active) gamers and on either playing traditional non-active games or active games rather than both types of gaming simultaneously. The latter is necessary to examine differences in the correlates of active gaming and non-active gaming. Moreover, no attention has been paid to potential personal, social, or game-related correlates of playing both active and non-active games. More specifically, the focus has been on who is playing active and non-active games rather than on why adolescents may play these games. Understanding why people play active and non-active games could provide directions to future intervention strategies attempting to substitute active game play for non-active game play. Therefore, the current study focused on potential personal, social, and game-related correlates of active as well as non-active gaming.

The selection of potential correlates for the current study was based on the findings of a focus group study conducted with adolescents about active and non-active gaming, which showed image, ease of use, and playing with others are important factors in gaming for adolescents [14]. Furthermore, the correlates were based on theories that have been applied to sedentary behavior, physical activity, or specific types of gaming [24-26]; Theory of Planned Behavior (TPB; attitude, descriptive norm) [27], Self-Determination Theory (SDT; autonomous motivation, game engagement) [28], Technology Acceptance Model (TAM; ease of use, competence) [29], and Habit Theory (habit) [30]. This has necessarily led to an extensive set of potential correlates tested, which we found appropriate given the fact that this is a first exploratory study of such correlates. The results can and will be used for more targeted exploration of correlates and potential determinants in future research. The correlates were structured according to three categories. First, personal correlates were distinguished, which refer to individual psychological factors such as attitude toward playing active and non-active games, autonomous motivation for playing video games, self-perceived gaming competence, habit regarding playing video games, and attitude toward physical activity. Second, we distinguished factors that relate to the social aspects of gaming (eg, descriptive norm) and social images of active and non-active gamers, referring to these as social correlates. Third, and finally, factors that are related to the video games were assessed, namely, perceived ease of use of playing active and non-active games, game engagement, and number of active and non-active games in possession.

The current study aimed to (1) examine potential personal, social, and game-related correlates of active gaming (≥1 h/wk) in adolescents; (2) examine potential personal, social, and game-related correlates of non-active gaming (>7 h/wk) in adolescents; and (3) compare the correlates of active gaming (≥1 h/wk) with those of non-active gaming (>7 h/wk).

## Methods

### Design and Procedure

The current study makes use of data gathered in a larger prospective study on video games among adolescents and their parents (not published yet). The present study reports cross-sectional data from the first questionnaire completed by adolescents. The questionnaire was administered in the classroom under the supervision of the researcher and/or a teacher. On the day of the first survey, a researcher explained the goals and procedures of the study in the classroom. The researcher asked adolescents in a school class session whether they played video games (active and/or non-active games) at least once a week. Those who answered “yes” (further referred to as “gaming adolescents”) and were willing to participate received the “gaming questionnaire”, containing questions about gaming characteristics and demographics. The adolescents who did not play video games received a “nongamers questionnaire”, consisting of questions about demographics. The adolescents received an information letter for their parents with a passive consent procedure indicating that parents could object to the study participation of their child. In such cases, the questionnaire of the corresponding child was destroyed. Among adolescents who completed the entire study, 2 MP3 players, 6 gift vouchers of €10 for video games, and 6 gift vouchers worth €25 for video games were raffled as an incentive.

### Participants

The Dutch secondary school system consists of three levels of education: (1) pre-vocational, (2) higher continued education, and (3) pre-university. The participants were recruited from 5 secondary schools in the Netherlands, covering all educational levels, whereby a maximum of 4 classes per school were included. The aim was to establish a representative sample of enrolled schools covering a wide range of socioeconomic, ethnic, and geographic characteristics, as described in Simons et al [19]. Therefore, the approached schools varied with respect to location (urban/nonurban) and educational level. In total, 459 students from 18 classes were invited to participate. The current study focused on the gaming adolescents only, resulting in 357 (77.7%) eligible students. Three parents objected to their child’s participation, and 1 student was dismissed from class because of misbehavior and not filling out the questionnaires seriously. The data from these students were therefore excluded, resulting in approved responses from 353 adolescents.

The Central Committee on Research Involving Human Subjects in the Netherlands provided an exemption for this study to seek formal approval from the Medical Ethics Committee.

### Measures

#### Demographic Factors

Questions regarding birth date (for calculating age), gender, and educational level (pre-vocational, higher education, pre-university) were included in the questionnaire. Educational level was dichotomized into low level (pre-vocational) and high level (higher education and pre-university).

#### Outcome Measures

We focused on 2 outcome measures: (1) time spent active gaming, and (2) time spent non-active gaming. To assess time spent active and non-active gaming, questions about frequency and duration were formulated based on existing and validated questionnaires for adolescents [31,32] for school and weekend days separately. Adolescents could indicate duration by selecting 1 of 4 categories (<30 minutes, 30 to <60 minutes, 1-2 hours, and >2 hours). The terms active and non-active games were explained as follows: non-active games are games in which players only have to use their fingers or hands, and active games are games that require movement of the body, more than only fingers and hands (eg, Nintendo Wii, PlayStation Move, Microsoft Kinect).

Time spent active gaming was dichotomized into durations lasting <1 h/wk and ≥1 h/wk [19,20]. Because there is no general accepted cutoff value for active gaming, this cutoff value was based on calculations in adults demonstrating that excessive weight gain can be prevented if energy balance is affected by 70 kcal/wk [21]. Based on calculations of energy expenditure during active video game play [15], substituting sedentary activities with playing active games for 1 h/wk corresponds to an additional 70 kcal of energy expenditure each week and may thus prevent excessive weight gain [20]. Second, 1 h/wk seems feasible to incorporate into intervention programs as described in the introduction.

Time spent non-active gaming was dichotomized into durations lasting ≤7 h/wk and durations lasting >7 h/wk. There is no general accepted cutoff value for non-active gaming. We used 1 hour per day as the cutoff value because this represents half of the 2 hours of maximum total screen time recommended for adolescents [22] and is the same cutoff value used by Allahverdipour et al in their study on non-active gaming [23].

Attitude, descriptive norm, image, perceived ease of use, and number of games in possession were assessed with respect to active and non-active gaming separately. Autonomous motivation, self-perceived gaming competence, habit, and game engagement were assessed with respect to gaming in general.

#### Personal Correlates

##### Attitude Toward Playing Active/Non-Active Games

Attitude (based on TPB [27]) was assessed by asking respondents to evaluate playing active/non-active games on six 5-point bipolar scales, based on a manual for constructing questionnaires based on TPB [33] (eg, “I think playing active/non-active games is”: “very stupid” [score of 1] to “very enjoyable” [score of 5]). The 6 items were combined into one construct by averaging the scores (attitude active gaming, Cronbach alpha=0.77; attitude non-active gaming, Cronbach alpha=0.73).

##### Autonomous Motivation for Playing Video Games

Type of motivation was deduced from the SDT [24,28] and assessed using a modified version of the Perceived Locus of Causality scale [34]. To prevent the questionnaire from becoming too lengthy, we used a modified version by selecting the 2 most relevant items with the highest factor loadings for each type of motivation [35]. Four types of motivation were assessed: (1) external regulation, (2) interjected regulation, (3) identified regulation, and (4) intrinsic regulation [34]. The respondents were asked to indicate on a 5-point scale (totally disagree [score of 1] to- totally agree [score of 5]) whether they agreed on statements starting with “I play video games …” for example, for interjected regulation, “because I want my friends to think that I am good in playing video games”; for identified regulation, “because I want to improve in playing video games”; and for intrinsic regulation, “because playing videogames is fun.” The 10 items were combined into a Relative Autonomy Index (RAI) by weighting the external subscale −2, the interjected subscale −1, the identified subscale +1, and the intrinsic subscale +2. Amotivation was not considered in the formulation of the RAI [36]. The minimum score for the RAI is −30, and the maximum score is +30. Higher positive scores for the RAI indicate more autonomous motivation, whereas lower negative scores indicate less autonomous motivation.

##### Self-Perceived Gaming Competence

Perceived competence is based on SDT, which contends that competence is one of the basic needs that drive behavior [24]. Self-perceived gaming competence was measured using the 3 most relevant items of the validated Intrinsic Motivation Inventory (originally consisting of 6 items) [37]. Respondents had to indicate on a 5-point scale ([score of 1] totally disagree to [score of 5] totally agree) whether they agreed with the following statements: “I believe I am good at playing video games”, “I think I am better at playing video games than other people my age and gender”, and “I am generally happy with my gaming performance.” The statements were combined into one construct (Cronbach alpha, 0.84). The Cronbach alpha in the current study was 0.84, which is comparable to the value of 0.81 that Markland and Hardy [38] observed for the competence subscale when they assessed the factorial and construct validity of the Intrinsic Motivation Inventory.

##### Habit Regarding Playing Video Games

To assess habit strength with respect to playing video games, we used 4 items from the 12-item Self-Reported Habit Index [30]. Four items that reflected 2 important aspects of habits were selected: automaticity (the extent to which particular behaviors are executed efficiently, outside control and awareness) and identity (the extent to which the behavior is part of everyday life and reflects a sense of personal style). The following items were included in the current study regarding playing video games (“Playing video games is something”): “…I do automatically” (automaticity), “…I start doing before I realize I'm doing it” (automaticity), “that is typically me” (identity), and “that belongs to my daily routine” (identity). Respondents were asked to indicate on a 5-point scale (totally disagree [score of 1] to totally agree [score of 5]) whether they agreed with the items. The reduced scale demonstrated good internal consistency in the current study (Cronbach alpha, 0.81).

##### Attitude Toward Physical Activity

Attitude toward physical activity was measured by asking respondents to evaluate physical activity on two 5-point bipolar scales, based on the manual for TBP questionnaires [33] (“Do you think it is fun or stupid to increase your physical activity behavior (very stupid [score of 1] to a lot of fun [score of 5])” and “Do you think it is good or bad to increase your physical activity behavior?” (very bad [score of 1] to very good [score of 5]). These 2 items were combined into one construct (Cronbach alpha, 0.76).

#### Social Correlates

##### Descriptive Norm Active and Non-Active Gaming

Descriptive norm (based on TPB [27]) was assessed on a scale from 1 (very little time) to 5 (very long time) with the following items, based on the TBP manual [33]: “Do most of your friends spend a lot of or little time playing active/non-active games?” and “Do your brothers or sisters spend a lot of or little time playing active/non-active games?” The items were dichotomized into low (score 1-3) and high descriptive norms (score 4-5) for brothers/sisters and friends separately.

##### Image Regarding Active Gamers and Non-Active Gamers

Image as a potential correlate for gaming arose from focus groups held with adolescents about active and non-active gaming [14]. Social image or prototype is also a construct belonging to the Prototype Willingness model [39] and denotes the image that an adolescent associates with a behavior or the perceptions of the type of person who performs the behavior [39]. The Prototype Willingness model was originally developed to explain health risk behaviors (eg, drinking and smoking) in adolescents and young adults, and studies have shown that the images adolescents hold of peers who engage in risk behaviors are associated with adolescents’ willingness to engage in risk behaviors when the opportunity arises [40,41]. To the best of our knowledge, the Prototype Willingness model has not yet been applied to gaming behavior. To assess the image of active and non-active gamers, respondents were asked to indicate what they thought of “an active/non-active gamer” using 6 characteristics. Respondents had to indicate on a 5-point scale ([score of 1] totally disagree to [score of 5] totally agree) whether they agreed with the following statements: “I think an active/non-active gamer is (1) unsportsmanlike, (2) cool, (3) childish, (4) companionable, (5) boring, or (6) attractive. Negative characteristics (unsportsmanlike, childish, and boring) were reversed, and statements were then combined into one construct for active gamers (Cronbach alpha, 0.62) and one construct for non-active gamers (Cronbach alpha, 0.77).

#### Game-Related Correlates

##### Perceived Ease of Use of Playing Active/Non-Active Games

Perceived ease of use is a construct deduced from the TAM and measures the extent to which people believe playing active/non-active games is effortless. To assess the perceived ease of use of playing active and non-active games, 2 questions were asked for active and non-active games separately: (1) “It is easy for me to learn how active/non-active games work”, and (2) “Playing active/non-active games is easy for me.” Respondents had to indicate on a 5-point scale ([score of 1] totally disagree to [score of 5] totally agree) whether they agreed. The 2 items were derived from Hsu and Lu [29] and were based on a validated questionnaire developed by Davis [42]. The 2 items were combined into one construct for non-active games (Cronbach alpha, 0.78) and one for active games (Cronbach alpha, 0.88).

##### Game Engagement

Game engagement is a generic indicator of game involvement that consists of the categories absorption, flow, presence, and immersion. Engagement was measured by means of the Game Engagement Questionnaire (GEQ) [43], which was developed to measure the potential of an individual to become engaged in video games. The GEQ consists of 19 items (eg, “If I play video games, I lose track of time”, “If I play video games, I do not hear if someone is talking to me”, “If I play video games, the game feels real”). Respondents had to answer on a 3-point scale (no [score of 1], a little [score of 2], yes [score of 3]), and the scores were summed to yield a cumulative score. The Cronbach alpha in the current study was 0.91, which is comparable to the value of 0.85 observed by Brockmeyer et al when developing the scale [43].

##### Number of Active and Non-Active Games in Possession

Respondents were asked to indicate how many active and non-active games they had in their household.

### Analyses

Of the 353 adolescents, 44 had missing values for one of the dependent variables (time spent active/non-active gaming), and another 59 adolescents had missing values for one of the potential correlates, resulting in 250 (80.9%) adolescents with complete data. We therefore decided to impute data using chained imputations [44] with an imputation model consisting of all the potential predictors, the dependent variables, and 6 other variables that we thought were related to missingness. These 6 variables were (1) spending a lot or a little time on playing active games (a little [score of 1] to a lot [score of 5]), (2) spending a lot or a little time on playing non-active games (a little [score of 1] to a lot [score of 5]), (3) playing active games in comparison with others (much less [score of 1] to much more [score of 5], (4) playing non-active games in comparison with others (much less [score of 1] to much more [score of 5]), (5) attitude toward spending more time on active gaming (scale 1-5), (6) attitude toward spending less time on non-active gaming (scale 1-5). Trace plots of means and standard deviations of imputed variables were checked for convergence. It was found that results were stable after 50 imputations, which was used in the final analyses.

Based on these 50 imputed databases, first descriptive analyses were performed on demographics to describe the study population and to explore personal, social, and game-related factors among gaming adolescents. Furthermore, to examine potential personal and game-related correlates of active ≥1 h/wk and non-active gaming >7 h/wk, 2 multilevel logistic regressions were performed, with all variables entered simultaneously. Variables entered in the model included all 17 potential correlates and the demographics (age, sex, and educational level of adolescents) that were observed to correlate with either active or non-active gaming [19]. We fitted multilevel models to correct for a potential clustering effect at the school and class levels. In the first multiple logistic regression, the dependent variable was active gaming for more or less than 1 h/wk. In the second multiple logistic regression, the dependent variable was non-active gaming for more or less than 7 h/wk. *P* values <.05 were considered to be statistically significant. The multiple imputations as well as all the analyses based on the imputed datasets were performed in STATA/SE 12.1. Finally, Spearman’s correlation coefficients on the complete cases were calculated to provide insight into relations between active gaming ≥1 h/wk and non-active gaming >7 h/wk and their potential correlates. This was done in IBM SPSS Statistics version 20.

## Results

### Participants

The mean age of the total group of participants (n=353) was 13.9 years (SD1.5); the majority were male (60.6%; 214/353) and attended a high level of education (64.6%; 228/353). Of the 353 participants, 33.2% (117/353) played active games ≥1 h/wk, 33.2% (117/353) played non-active games >7 h/wk, and 9.9% (35/353) played both active games ≥1 h/wk and non-active games >7 h/wk.

Table 1 shows the descriptive statistics for the potential correlates for active (≥1 h/wk) and non-active gamers (>7 h/wk) separately. In general, participants had a positive attitude toward both active and non-active gaming and had friends who spent much time on non-active gaming. Furthermore, the participants had a positive attitude toward PA and thought the use of active and non-active games was easy.

**Table 1:** Means and standard deviations of potential correlates of active and non-active gaming (N=353).

Characteristic (scale)		Active gaming (h/wk)		Non-active gaming (h/wk)	
		Active gamers (≥1)	Active gamers (<1)	Non-active gamers (>7)	Non-active gamers (≤7)
**Personal correlates, mean (SD)** ^a^					
	Attitude toward playing active games (1-5)	3.5 (0.79)	3.1 (0.8)	3.1 (1.3)	3.3 (0.6)
	Attitude toward playing non-active games (1-5)	3.3 (1.0)	3.4 (0.8)	3.8 (0.9)	3.2 (0.6)
	Autonomous motivation for playing video games (−30 through +30)	3.8 (4.4)	4.1 (3.2)	4.5 (4.3)	3.7 (3.1)
	Self-perceived gaming competence (1-5)	3.2 (1.8)	3.3 (1.2)	3.8 (1.6)	3.0 (1.1)
	Habit regarding playing video games (1-5)	2.7 (1.9)	2.6 (1.2)	3.5 (4.3)	2.3 (1.0)
	Attitude toward physical activity (1-5)	4.2 (1.4)	4.3 (0.9)	4.0 (1.3)	4.4 (0.8)
**Social correlates**					
	Descriptive norm active gaming of *…* friends (% high score on descriptive norm scale)	21	7	10	13
	Descriptive norm active gaming of *…* brothers/sisters (% high score on descriptive norm scale)	29	7	11	16
	Descriptive norm non-active gaming of … friends (% high score on descriptive norm scale)	51	47	76	35
	Descriptive norm non-active gaming of … brothers/sisters (% high score on descriptive norm scale)	31	34	32	33
	Image regarding active gamers (1-5; mean, SD)	3.2 (0.9)	3.1 (0.7)	3.0 (1.0)	3.2 (0.6)
	Image regarding non-active gamers (1-5; mean, SD)	3.3 (1.4)	3.4 (1.0)	3.8 (1.2)	3.2 (0.9)
**Game-related correlates, mean (SD)**					
	Perceived ease of use of playing active games (1-5)	3.9 (1.9)	3.7 (1.3)	3.7 (2.1)	3.8 (1.2)
	Perceived ease of use of playing non-active games (1-5)	4.1 (1.7)	4.1 (1.1)	4.3 (1.6)	4.0 (1.1)
	Game engagement (19-57)	31.1 (16.8)	31.0 (10.8)	35.6 (15.7)	28.7 (9.9)
	Number of active games in possession	12.1 (61.7)	3.2 (15.3)	9.2 (14.7)	4.7 (14.7)
	Number of non-active games in possession	37.5 (116.7)	35.8 (68.6)	54.5 (118.5)	27.3 (66.2)

^a^Mean and SD are based on results from 50 imputation for the missing values.

### Correlates of Active Gaming

Next, we evaluated which factors correlated with active (≥1 h/wk) and non-active gaming (>7 h/wk) in multivariable analyses. The regression analyses revealed the following statistically significant correlates for active gaming (≥1 h/wk; Table 2): Personal: “attitude toward active gaming”, “attitude toward non-active gaming”, and “habit regarding playing video games.” Social: “descriptive norm active gaming of friends”, and “descriptive norm active gaming of brothers/sisters.” Game-related: “game engagement.” Active gamers (≥1 h/wk) had a more positive attitude toward active gaming, a less positive attitude towards non-active gaming, a higher score on habit strength regarding gaming, had brothers/sisters and friends who spend more time on active gaming, and scored lower on game engagement.

### Correlates of Non-Active Gaming

With respect to non-active gaming (>7 h/wk), the statistically significant correlates were Personal: “attitude toward non-active games” and “habit regarding playing video games.” Social: “descriptive norm non-active gaming of friends”, and “image regarding non-active gamers.” None of the game-related correlates were significant. Non-active gamers (>7 h/wk) had a more positive attitude toward non-active games, had a higher score on habit strength regarding gaming, had friends who spend more time on non-active gaming, and a more positive image regarding non-active gamers.

**Table 2:** Logistic regression analyses of correlates of active ≥1 h/wk and non-active gaming >7 h/wk.

Correlate^a^		Active gaming ≥1 h/wk^b^			Non-active gaming >7 h/wk^b^		
		OR	95% CI	*P* value	OR	95% CI	*P* value
**Personal**							
	Attitude toward active gaming^c^	5.3	2.4-11.8	<.001	0.5	0.23-1.0	.052
	Attitude toward non-active gaming^c,d^	0.3	0.1-0.6	.002	2.6	1.1-6.3	.035
	Autonomous motivation for playing video games	1.0	0.8-1.1	.58	1.1	0.96-1.3	.14
	Self-perceived gaming competence	1.0	0.6-1.5	.88	0.84	0.51-1.37	.49
	Habit regarding gaming^c,d^	1.9	1.2-3.2	.008	3.0	1.7-5.3	<.001
	Attitude toward physical activity	0.9	0.5-1.5	.7	0.8	0.5-1.3	.35
**Social**							
	Descriptive norm active gaming: friends^c^	3.4	1.4-8.4	.0089	0.57	0.20-1.65	.30
	Descriptive norm active gaming: brothers/sisters^c^	6.7	2.6-17.1	<.001	0.54	0.17-1.74	.30
	Descriptive norm non-active gaming: friends^d^	1.3	0.6 -2.6	.55	3.3	1.46-7.53	.004
	Descriptive norm non-active gaming: brothers/sisters	0.7	0.3-1.3	.24	0.6	0.27-1.33	.21
	Image regarding active gamers	1.3	0.6-2.8	.58	0.51	0.21-1.23	.13
	Image regarding non-active gamers^d^	0.9	0.5-1.5	.6	2.0	1.07-3.75	.030
**Game-related**							
	Perceived ease of use of playing active games	1.2	0.8-1.7	.31	0.94	0.64-1.39	.75
	Perceived ease of use of playing non-active games	0.9	0.6-1.3	.49	1.24	0.78-1.96	.36
	Game engagement^c^	0.95	0.91-0.997	.04	1.02	0.97-1.08	.37
	Number of active games owned	1.03	0.99-1.07	.098	1.03	0.99-1.06	.13
	Number of non-active games owned	1.0	0.99-1.0	.78	1.0	0.99-1.0	.65

^a^Adjusted for demographics: gender, age, educational level.

^b^Values are shown based on results from 50 imputation for the missing values (N=353).

^c^Significant correlate for active gaming ≥1 h/wk.

^d^Significant correlate for non-active gaming >7 h/wk.

### Bivariate Associations

Table 3 presents the bivariate correlations between the potential correlates and active (≥1 h/wk) and non-active gaming (>7 h/wk) based on the complete case sample (N=250). Active gaming (≥1 h/wk) was significantly, strongly, positively correlated (*r*≥0.5) with the number of active games owned; significantly, moderately, positively correlated (*r=*0.3) with attitude toward non-active gaming and descriptive norm active gaming brothers/sisters; and significantly, weakly, positively correlated (*r*=0.1) with descriptive norm active gaming friends, image regarding active gamers, and perceived ease of use of active games.

Non-active gaming (>7 h/wk) was significantly, strongly, positively correlated (*r*≥0.5) with attitude toward non-active gaming, habit, and image regarding non-active games and significantly moderately, positively correlated (*r*=0.3) with self-perceived gaming competence, descriptive norm non-active gaming friends, perceived ease of use of non-active gaming, game engagement, and number of non-active games owned. A significant, moderate, negative association was observed with attitude toward physical activity. The highest correlation coefficient among the correlates was 0.58, indicating that colinearity is not a problem.

**Table 3:** Spearman’s correlations between active gaming ≥1 h/wk, non-active gaming >7 h/wk, and potential correlates based on the complete case sample (n=250).

	2	3	4	5	6	7	8	9	10	11	12	13	14	15	16	17	18	19
1.Active gaming ≥1 h/wk	−.03	.34^c^	−.11	-.03	−.02	.04	−.01	.21^c^	.30^c^	.03	−.03	.17^b^	−.02	.18^b^	.003	−.01	.54^c^	.01
2.Non-active gaming >7 h/wk	–	−.09	.49^c^	.12	.41^c^	.58^c^	−.24^c^	−.04	−.07	.38^c^	−.02	−.06	.49^c^	−.02	.25^c^	.38^c^	−.07	.30^c^
3.Attitude towards active gaming		–	.06	.13^a^	.03	.05	.03	.10	.07	.06	.0	.36^c^	−.03	.27^c^	.11	.08	.42^c^	.07
4.Attitude toward non-active gaming			–	.26^c^	.50^c^	.53^c^	−.18^b^	.14^a^	−.12	.35^c^	.02	.05	.50^c^	.12	.38^c^	.39^c^	−.09	.37^c^
5.Autonomous motivation for playing video games				–	0.26^c^	.03	.06	.07	.07	.30^c^	.11	−.01	.16^a^	.11	.17^b^	.21^c^	.04	.12
6.Self-perceived gaming competence					–	.53^c^	−.13^a^	.03	.04	.34^c^	.0	−.0	.49^c^	.02	.37^c^	.40^c^	.0	.29^c^
7.Habit regarding gaming						–	-.22^c^	.02	−.10	.37^c^	.05	.03	.54^c^	−.06	.24^c^	.54^c^	−.03	.36^c^
8.Attitude toward physical activity							–	.05	.11	−.09	.08	.01	−.12	.01	−.04	−.21 ^c^	.05	−.08
9.Descriptive norm active gaming friends								–	.17^b^	.15^a^	−.01	.14^a^	.05	.02	−.02	.09	.06	−.04
10.Descriptive norm active gaming brothers/sisters									–	−.06	.18^b^	−.01	−.07	.09	.10	.04	.32^c^	.08
11.Descriptive norm non-active gaming friends										–	.09	−.04	.37^c^	.02	.10	.30^c^	.05	.21^c^
12.Descriptive non-active gaming brothers/sisters											–	.03	−.02	.09	.02	.19^b^	.09	.08
13.Image regarding active gamers												–	.16^b^	.08	.04	.13^a^	.18^b^	.03
14.Image regarding non-active games													–	−.04	.25^c^	.37^c^	−.04	.39^c^
15.Ease of use active gaming														–	.42^c^	−.002	.16^a^	.10
16.Ease of use non-active gaming															–	.22^c^	.03	.27^c^
17.Game engagement																–	−.0	.28^c^
18.Number of active games owned																	–	.25^c^
19.Number of non-active games owned																		–

^a^Correlation is significant at the .05 level (2-tailed).

^b^Correlation is significant at the .01 level (2-tailed).

^c^Correlation is significant at the .001 level (2-tailed).

## Discussion

### Overview

The aim of the present study was to examine correlates of active gaming ≥1 h/wk and non-active gaming >7 h/wk and to compare these correlates, taking demographics such as gender, age, and educational level into account. A greater understanding of these correlates contributes to understanding why people play active and non-active games and provides insight into potential barriers of and opportunities for intervention strategies attempting to substitute active game play for non-active game play.

The findings of the present study show that significant correlates of active gaming ≥1 h/wk include the personal factors attitude toward active gaming, attitude toward non-active gaming, habit regarding gaming, the social factors descriptive norm active gaming of brothers/sisters and friends, and the game-related factor game engagement (first research aim). Significant correlates of non-active gaming >7 h/wk include the personal factors attitude toward non-active gaming and habit regarding gaming, and the social factor descriptive norm non-active gaming of friends and image of non-active gamers (second research aim). When comparing correlates of active gaming with non-active gaming, it shows that attitude toward non-active gaming (although the direction differs), habit strength, and descriptive norms (active or non-active gaming) of friends are the only factors associated with both types of gaming (third research aim).

### Sole Correlates of Active Gaming

Important correlate of active gaming ≥1 h/wk included the social factor descriptive norm active gaming of brothers/sisters, in line with previous studies that showed that the social aspect was important for ongoing participation in playing active games [14,45,46]. Furthermore, observational real life studies showed that active games are often played with siblings [19,47]. Because descriptive norm active gaming of brothers and sisters was the most important correlate for active gaming ≥1 h/wk, we recommend that active game intervention strategies focus on families instead of individuals.

Game engagement was a weak correlate for active gaming. Active gamers were a bit less likely to be engaged during playing games. Game engagement was measured with the validated GEQ, a questionnaire to measure the potential of an individual to become engaged in video games [43]. However, based on this study, we do not know if it refers to a trait in the sense that some adolescents become more easily immersed when gaming or a state in the sense that some games have stronger immersive qualities (or a mix).

A commonly expressed concern about active games is that only youth who like physical activity and are already physically active (and therefore not a target group for health promotion interventions) will play active games. However, the findings of the current study do not support this concern because we found that attitude with respect to physical activity was unrelated to active game play ≥1 h/wk.

### Sole Correlates of Non-Active Gaming

Image regarding non-active gamers was the only factor that solely correlated with non-active gaming. Adolescents playing non-active games >7 h/wk were more positive about the image of a non-active gamer. Image or prototype is a construct belonging to the Prototype Willingness model [39] and denotes the image that an adolescent associates with a behavior or the perceptions of the type of person who performs the behavior (in this case non-active gaming) [39]. The Prototype Willingness model has mainly been applied to risk behaviors such as drinking and smoking; to our knowledge, it has not been applied yet to gaming behavior. Image was mentioned as a factor during focus groups with adolescents about gaming, but only regarding active gaming [14,48]. Some New Zealand girls (10-12 years old) did not see themselves playing active games once they reached high school, because it could be embarrassing. They thought playing active games is less socially acceptable for older girls than for younger girls [48]. In focus groups with Dutch adolescents, it was mentioned that it was not “cool” to play active games on your own. However, in the current quantitative study, these findings were not confirmed, because image appeared only to be a correlate for non-active gaming and not for active gaming.

### Comparison of Correlates of Active and Non-Active Gaming

Attitude appeared to be an important personal correlate for both active and non-active gaming. With respect to active gaming, on one hand, active gamers (≥1 h/wk) had a more positive attitude toward active gaming than adolescents who play active games <1 h/wk. This result suggests that it is important that adolescents have a positive attitude toward active gaming when aiming to replace non-active games with active games. On the other hand, we found that attitude toward non-active games was strongly negatively associated with active gaming ≥1 h/wk. Because of the cross-sectional design of the study, we do not know whether becoming an active gamer for ≥1 h/wk results in becoming less positive about non-active games or whether adolescents who are less positive about non-active games are more likely to turn to active games. The first could be positive for intervention strategies aiming at replacing non-active games with active ones. However, it might also be true that the more adolescents enjoy and favor non-active games, and thus form an important target group, the less likely they are to replace their non-active game play with active games, and therefore form a target group that is difficult to reach. Attitude toward non-active games was also a correlate for non-active gaming >7 h/wk. Attitude toward non-active gaming was negatively associated with active gaming ≥1 h/wk and positively associated with non-active gaming >7 h/wk, which suggests it might be difficult to transform non-active gamers into active gamers.

Enjoyment is an important element of attitude, and intervention strategies should therefore consider the aspects that adolescents like about active games, namely, being physically active, interactivity, realistic body movements, one-to-one translation of their movements into the game, and playing with other people [14]. Although studies have shown that many adolescents enjoy playing active games, in the long term, boredom often strikes and use declines over time [14,15,46]. The aspects of video games that make them attractive in the long term are online modus, multiplayer options, and the opportunity to improve oneself [14]. Lyons et al showed that multiplayer options were prevalent in half of the 18 evaluated active games [49]. The most prevalent behavioral strategy was performance feedback (in 17 of the 18 active games), which opens up the opportunity to improve oneself. To ensure long-term enjoyment in active gaming, it is important that more active games include one of these features and that game developers develop more active games that remain enjoyable in the long run.

Habit strength was associated with both active ≥1 h/wk as well as non-active gaming >7 h/wk, suggesting that playing both types of gaming is a habitual activity. This is an interesting finding for future interventions targeting game behavior because habitual behaviors may be more difficult to change and require different strategies than nonhabitual behaviors [50]. For example, intervention strategies based on information provision might not be effective because the habitual behavior (gaming) may override the attentional mechanisms needed to process such information [51,52]. Habits are triggered by situational and environmental cues; therefore, behavior change strategies should focus on incorporating environmental cues [53]. For strategies aimed at replacing non-active games with active games, one may consider placing the active game console in a highly visible place so that it can serve as a cue for playing. On the other hand, the non-active game console should be placed in a less visible place to prevent it from serving as a cue for playing. Findings from a focus group study confirm that seeing an active game console serves as a cue for playing it [14].

Descriptive norm non-active gaming of friends was a correlate of non-active gaming >7 h/wk. Adding the finding that descriptive norm active gaming of friends was a correlate for active gaming ≥1 h/wk makes modeling behavior of friends an important factor for game behavior. Remarkably, non-active game behavior of brothers and sisters was not associated with non-active gaming >7 h/wk, because active game behavior of brothers and sisters was for active gaming ≥1 h/wk.

### Limitations and Strengths

The present study is subject to some limitations that need to be acknowledged. First, the cross-sectional design precludes any inferences from being made about causal mechanisms. Second, all measures were based on self-reported information, which may suffer from recall bias and socially desirable answers. Although we based the measures upon readily existing instruments, some of the scales had to be shortened to avoid a lengthy questionnaire. Doing so might have influenced the validity of the included measures. However, if a scale was shortened, we removed the items with the lowest factor loading, minimizing the possible negative influence on validity. Nevertheless, the use of selected items from validated scales is a limitation of the present study. Furthermore, we chose cutoff values for active and non-active gaming of 1 and 7 h/wk, respectively, which is arbitrary. There are no recommendations for the maximum time spent playing video games; therefore, we based our cutoff values on the results of previous studies and calculations for recovering the energy imbalance estimations [19-21,23].

One important strength of the current study is that it is the first to compare correlates of both active and non-active gaming. Furthermore, we included a wide range of personal, social, and game-related variables, which were based on behavioral theories and the outcomes of focus groups. The current study provides important new insights into personal, social, and game-related correlates of both active and non-active gaming.

### Conclusions

Various factors were significantly associated with active gaming ≥1 h/wk and non-active gaming >7 h/wk. Active gaming is most strongly (negatively) associated with attitude with respect to non-active games, followed by observed active game behavior of brothers and sisters, and attitude with respect to active gaming (positive associations). On the other hand, non-active gaming is most strongly associated with observed non-active game behavior of friends, habit strength regarding gaming, and attitude toward non-active gaming (positive associations). Habit strength was a correlate of both active and non-active gaming, indicating that both types of gaming are habitual behaviors. Although these results should be interpreted with caution because of the limitations of the study, they do provide preliminary insights in potential correlates of active and non-active gaming, which can be used for further research as well as preliminary direction for the development of effective intervention strategies for replacing non-active gaming by active gaming among adolescents.

## Abbreviations

GEQ: game engagement questionnaire
h/wk: hours per week
PA: physical activity
RAI: relative autonomy index
SDT: self-determination theory
TAM: technology acceptance model
TPB: theory of planned behavior
